# Atlanto-Occipital Dissociation With Catastrophic Neurological Injury: Ethical Challenges in the Intensive Care Setting

**DOI:** 10.7759/cureus.99105

**Published:** 2025-12-13

**Authors:** Rita Almeida, Nuno Gatta, José Manuel Pereira, José-Artur Paiva

**Affiliations:** 1 Intensive Care Department, Unidade Local de Saúde de Viseu Dão-Lafões, Viseu, PRT; 2 Intensive Care Department, Unidade Local de Saúde de São João, Porto, PRT; 3 Intensive Care Medicine Department, Centro Hospitalar Universitário de São João, Porto, PRT; 4 Faculty of Medicine, Department of Medicine, University of Porto, Porto, PRT

**Keywords:** atlanto-occipital dissociation, cervical trauma, critical care, ethical consideration, traumatic brain injury

## Abstract

Atlanto-occipital dissociation (AOD) is a rare but severe cervical injury usually caused by high-energy trauma and often fatal before hospital admission. Advances in prehospital care have led to more patients surviving long enough for diagnosis, although their prognosis remains poor. We report a 42-year-old man who fell from approximately 5 meters, resulting in cardiorespiratory arrest at the scene. After achieving return of spontaneous circulation, imaging revealed AOD along with severe traumatic brain injury and widespread brainstem damage. Given the absence of neurological viability and the severity of his injuries, no surgical procedures were performed. The patient was admitted to the intensive care unit as a potential organ donor. This case highlights the diagnostic and ethical challenges caused by AOD in critical care. Early recognition of this severe injury is crucial, but clinicians must also face complex decisions regarding therapeutic futility and organ donation. An interdisciplinary, evidence-based, and ethically grounded approach remains essential in managing such patients. AOD remains a rare but devastating injury with extremely poor outcomes. This case highlights the importance of early suspicion, accurate diagnosis, and ethically guided decision-making in critical care.

## Introduction

Atlanto-occipital dissociation (AOD) is a rare but severe ligamentous injury, typically resulting from high-energy cervical trauma and often associated with head or spinal cord injury [1,2]. Although rare, it is regarded as one of the most deadly cervical spine injuries, with many patients dying on the scene or during transport [3].

AOD involves disruption of the atlanto-occipital joint, the articulation between the occipital condyles of the skull and the atlas (C1). This joint offers stability and controlled motion at the craniovertebral junction. First described in 1908, this injury should always be suspected in the context of high-energy trauma and is frequently associated with other serious injuries, especially head and spinal cord injuries [3,4].

AOD should be considered when trauma mechanisms involve hyperextension, hyperflexion, or lateral flexion of the cervical spine, as the combination of these movements markedly increases the likelihood of injury [3]. Specifically, it can be classified into three types according to the direction of displacement: type I, anterior translation of the occiput relative to the atlas; type II, longitudinal (traction) separation; and type III, posterior displacement [2].

AOD is one of the most serious cervical spine injuries, with a high risk of neurological sequelae and death [4]. Clinical presentation is highly variable, and in up to 20% of cases, the only symptom may be neck pain [3,4]. Among patients who reach the hospital alive, presentations range from intact neurological function to complete or incomplete spinal cord injury [4]. The neurological status at hospital admission remains the most important prognostic factor for outcome in patients with suspected cervical or spinal cord trauma [4].

Although AOD remains associated with high mortality, advances in prehospital and emergency care have increased the likelihood of survival long enough for diagnosis and management [2,5].

We report a case of traumatic AOD associated with severe traumatic brain injury, illustrating diagnostic and ethical challenges in the intensive care setting.

## Case presentation

A 42-year-old male with a past medical history of dyslipidemia, allergic rhinitis, and anxiety - medicated accordingly and with no known drug allergies - was admitted to the emergency department (ED) after a fall from approximately 5 meters. 

The accident occurred while he was standing on a platform at work that suddenly collapsed. At the scene, the patient was found unconscious, and bystanders initiated basic life support. Upon arrival of the prehospital team, the patient was in cardiorespiratory arrest, and advanced life support (ALS) was initiated. Spontaneous circulation returned (ROSC) after two cycles of ALS, both in a non-shockable rhythm. Airway patency was maintained with a laryngeal mask, and the patient was stabilized and transported to the hospital. The only drug used in the pre-hospital setting was fentanyl (and adrenaline during ALS). There was no neurological recovery after ROSC. 

On admission to the ED, neurological evaluation revealed a Glasgow Coma Scale (GCS) score of 3 and miotic pupils, with no additional abnormalities on physical examination. The patient was hemodynamically unstable, presenting with tachycardia and requiring norepinephrine infusion to maintain a mean arterial pressure above 65 mmHg. To secure the airway, the laryngeal mask was replaced by an endotracheal tube, with fentanyl administered as the sole induction agent.

**Figure 1:**
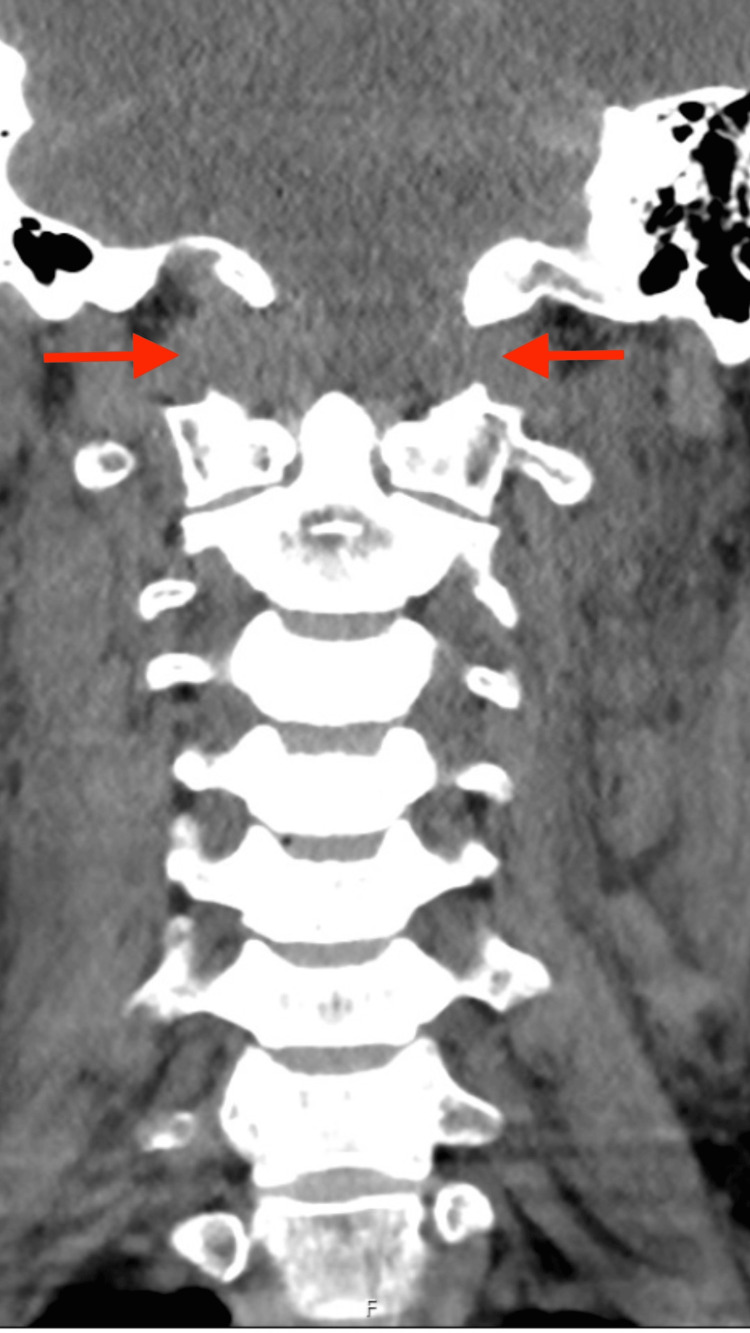
Coronal computed tomography (CT) reconstruction showing widening of the occiput–C1 joint space (arrows), consistent with atlanto-occipital dissociation.

**Figure 2:**
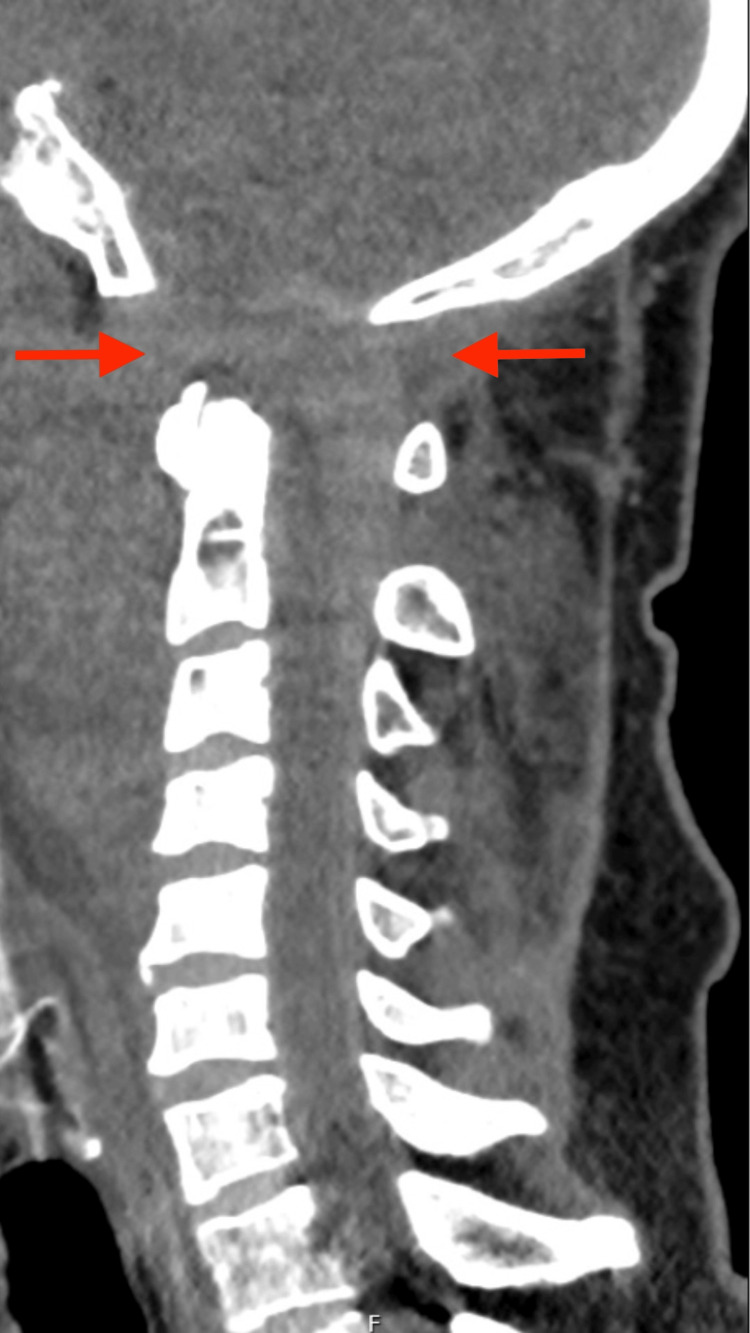
Sagittal computed tomography (CT) reconstruction showing widening of the occiput–C1 joint space (arrows), consistent with atlanto-occipital dissociation.

Imaging studies revealed severe traumatic lesions, most notably cervical trauma with AOD, characterized by approximately 2 cm of basion-odontoid separation and widening of the atlanto-occipital joint spaces (up to 1.2 cm), which are visible in both the coronal (Fig. 1) and sagittal (Fig. 2) computed tomography (CT) planes.

Concomitant, severe TBI was evident, with diffuse cisternal and sulcal subarachnoid hemorrhage, as well as intraventricular and tentorial subdural hemorrhages. This injury was associated with a large hematoma at the cranio-cervical junction causing deformation and contralateral displacement of the medullary cord, obliteration of cerebrospinal fluid spaces at the foramen magnum with caudal extension into the spinal canal, and diffuse vascular irregularities with caliber reduction of the main intracranial arterial axes, predominantly affecting the vertebrobasilar system, resulting in right pontine hypodensity extending into the middle cerebellar peduncle. Additional injuries included bilateral rib fractures without hemopneumothorax. No other traumatic findings were observed on the rest of the thoraco-abdomino-pelvic CT scan.

Given the absence of clinical signs of brainstem viability and the severity of the imaging findings, the patient was admitted to the intensive care unit as a probable donor. 

Written informed consent for publication was obtained from the patient’s legal representative.

## Discussion

AOD is a rare yet severe cervical injury, typically resulting from high-energy trauma. Its high mortality rate is mainly due to devastating neurological damage, leading many patients to die before reaching medical facilities. Nevertheless, improvements in prehospital care have increased survival rates for diagnosis and treatment, although the overall prognosis remains poor. At the same time, severe trauma cases often necessitate early ethical and prognostic discussions in the intensive care setting, especially when initial signs point to irreversible neurological injury.

In the case presented, the victim suffered significant trauma after a fall from approximately three times his height, requiring ALS at the scene. Upon arrival at the hospital, after stabilization and initial assessment, imaging confirmed not only severe TBI but also AOD. This finding emphasizes the strong link between this type of injury and other severe lesions, as it often occurs in high-energy traumatic mechanisms.

In recent decades, advances in prehospital and hospital care have significantly improved the management of polytraumatized patients, leading to better survival rates in cases that were previously often fatal. The use of trauma protocols based on Advanced Trauma Life Support (ATLS) principles has facilitated earlier detection of critical injuries, effective stabilization, and appropriate transfer to specialized centers. Furthermore, enhancements in cervical immobilization techniques at the scene have decreased the risk of secondary spinal injuries. Despite these developments, the prognosis of AOD still largely depends on the initial trauma severity and whether there is irreversible neurological damage, emphasizing the importance of a multidisciplinary, evidence-based approach.

Recent literature reflects this evolving landscape. Labler et al. reported several adult AOD survivors, noting that while still rare, their occurrences are increasingly documented [5]. Similarly, Prabhakar et al. observed higher survival rates in patients treated in recent years at a level-1 trauma center, indicating significant progress in trauma care [6]. More recent case reports detail survivors of traumatic AOD following prompt diagnosis and stabilization [7], and a current case series shows that survival until hospital admission - and even to definitive treatment - is now more common than previously recognized [8].

These findings align with our patient’s situation, who benefited from quick recognition and advanced trauma care, although the neurological injury was ultimately catastrophic.

Patients with severe traumatic brain injury also represent a significant group of potential organ donors. Trauma remains a substantial proportion of deceased donors worldwide, especially among younger adults. A recent multicenter study demonstrated that structured protocols-such as standardized neurological assessments and early notification of organ donation organizations-substantially decrease missed donation opportunities and enhance donor identification [9]. Additional ICU-focused research emphasizes that optimized hemodynamic support, early endocrine therapy, and coordinated multidisciplinary management improve organ viability after brain death, even in trauma-related cases [10-12]. These findings highlight how care has evolved for these patients, whose rapid progression to brain death ultimately allowed for organ donation consideration despite the lack of therapeutic options.

In the present case, aside from the severe TBI and AOD, the patient had no other major injuries. However, given the catastrophic and irreversible nature of the neurological damage, brain death was inevitable. The clinical presentation on admission, together with the imaging findings, was decisive in the decision not to pursue invasive surgical interventions. This situation exemplifies a common challenge in intensive care: determining when further treatment offers no realistic chance of neurological recovery and when continuation of invasive measures may constitute therapeutic futility.

Several prognostic factors have been associated with outcomes in AOD, such as high-energy trauma mechanisms, GCS at presentation, and associated brainstem or vascular injuries. Abbasi Fard et al. specifically found that concomitant traumatic brain injury markedly raises the risk of death in patients with atlanto-occipital dislocation, which aligns with the fatal progression seen in our patient [4].

This case emphasizes the importance of careful assessment in severe trauma situations, along with the ethical and clinical considerations needed when confronting therapeutic futility in intensive care. Treating catastrophic trauma goes beyond simply stabilizing the patient and conducting imaging; it also involves clear communication with the family, achieving consensus among healthcare teams on care objectives, and adhering to ethical standards for end-of-life decisions in the ICU.

## Conclusions

AOD is an uncommon but devastating injury, usually associated with high-energy trauma and high mortality rates.

The present case highlights the complexity of managing these patients, from quickly identifying the injury to making ethical and clinical decisions regarding therapeutic futility. It also emphasizes the importance of early suspicion of AOD in high-energy trauma cases and reinforces the need for a multidisciplinary approach to patient care.

The absence of neurological recovery after ROSC and the subsequent consideration of organ donation demonstrate the catastrophic nature of this presentation and illustrate the clinical and ethical challenges involved.

Despite major advances in prehospital and emergency care, the prognosis of AOD with severe neurological damage remains very poor, emphasizing the need to evaluate the proportionality of life-support interventions.
